# Increased efficacy of influenza virus vaccine candidate through display of recombinant neuraminidase on virus like particles

**DOI:** 10.3389/fimmu.2024.1425842

**Published:** 2024-06-10

**Authors:** Leticia Guzman Ruiz, Alexander M. Zollner, Irene Hoxie, Elsa Arcalis, Florian Krammer, Miriam Klausberger, Alois Jungbauer, Reingard Grabherr

**Affiliations:** ^1^Institute of Molecular Biotechnology (IMBT), Department of Biotechnology (DBT), University of Natural Resources and Life Sciences Vienna (BOKU), Vienna, Austria; ^2^Institute of Bioprocess Science and Engineering (IBSE), Department of Biotechnology (DBT), University of Natural Resources and Life Sciences Vienna (BOKU), Vienna, Austria; ^3^Department of Microbiology, Icahn School of Medicine at Mount Sinai, New York, NY, United States; ^4^Center for Vaccine Research and Pandemic Preparedness (C-VaRPP), Icahn School of Medicine at Mount Sinai, New York, NY, United States; ^5^Institute of Plant Biotechnology and Cell Biology (IPBT), Department of Applied Genetics and Cell Biology (DAGZ), University of Natural Resources and Life Sciences Vienna (BOKU), Vienna, Austria; ^6^Department of Pathology, Molecular and Cell-Based Medicine, Icahn School of Medicine at Mount Sinai, New York, NY, United States; ^7^Ignaz Semmelweis Institute, Interuniversity Institute for Infection Research, Medical University of Vienna, Vienna, Austria; ^8^Austrian Centre of Industrial Biotechnology (acib), Vienna, Austria

**Keywords:** virus like particle, neuraminidase, influenza, vaccine candidate, purification platform, unadjuvanted, gag-based

## Abstract

Vaccination against influenza virus can reduce the risk of influenza by 40% to 60%, they rely on the production of neutralizing antibodies specific to influenza hemagglutinin (HA) ignoring the neuraminidase (NA) as an important surface target. Vaccination with standardized NA concentration may offer broader and longer-lasting protection against influenza infection. In this regard, we aimed to compare the potency of a NA displayed on the surface of a VLP with a soluble NA. The baculovirus expression system (BEVS) and the novel virus-free Tnms42 insect cell line were used to express N2 NA on gag-based VLPs. To produce VLP immunogens with high levels of purity and concentration, a two-step chromatography purification process combined with ultracentrifugation was used. In a prime/boost vaccination scheme, mice vaccinated with 1 µg of the N2-VLPs were protected from mortality, while mice receiving the same dose of unadjuvanted NA in soluble form succumbed to the lethal infection. Moreover, NA inhibition assays and NA-ELISAs of pre-boost and pre-challenge sera confirm that the VLP preparation induced higher levels of NA-specific antibodies outperforming the soluble unadjuvanted NA.

## 1 Introduction

Vaccination offers a crucial defense against influenza virus infection and its possible complications. Every year the World Health Organization (WHO) recommends specific strains to be part of the influenza vaccines. Currently licensed vaccines are standardized solely in terms of the hemagglutinin (HA) content (1) and do not induce robust anti-neuraminidase (NA) immunity (2). The inclusion of standardized amounts of correctly folded NA either as soluble or included in a VLP may induce a more balanced immune response that also focuses on NA which may lead to a better antigenic match between vaccines and circulating viruses (3) and might potentially increase efficacy whenever the HA of the vaccine strain does not sufficiently resemble the virulent strain.

The WHO estimates that there are still approximately a billion cases of seasonal influenza each year, along with 3–5 million severe cases and around half a million deaths. Pregnant women, individuals with chronic illness, those receiving immunosuppressive treatments, the elderly (over 65 years of age), and young children (under 5 years of age) are the groups with the highest risk of developing severe disease or complications from an influenza A virus infection (4–6). HA and NA surface glycoproteins are the primary targets of protective antibodies and the most antigenic and highly variable influenza virus components (7–9). The degree of similarity between these proteins of circulating viruses and those included in the vaccines is an important element that determines the efficacy of influenza immunization (10, 11). Making efficient vaccines becomes a significant challenge when taking into account that the HA and NA surface antigens are constantly evolving to avoid the antibody-mediated immunity induced by infection or vaccination, which is commonly referred to as antigenic drift (12). Although, it has been highlighted that NA evolves independently from and to some degree slower than the HA (13, 14), current influenza virus vaccines so far have not considered the potential of broadening the immune reaction and protection (2, 15) by adding NA. In fact, several studies agree that influenza vaccination would be more effective and potentially produce broader and longer-lasting cross-protection by including a specified quantity of correctly folded NA (16–21). When adding NA to an existing vaccine, addition of extra adjuvants should be avoided, since increased reactogenicity could arise as a consequence. While soluble proteins often are inefficiently immunogenic when unadjuvanted, particle structures often induce immune response without the presence of adjuvanting agents (22).

Here, and based on previous work (22–24), we have generated VLPs in insect cells, that are based on self-assembly and budding of the human immunodeficiency virus 1 (HIV-1) gag protein, and display N2 NA on their surface. N2 was chosen because of the high diversity of H3N2 viruses in the human population and the resulting typically low vaccine effectiveness (25). These VLPs were expressed and purified using a platform downstream process based on Capto-Core 700™ flow-through chromatography followed by Capto-Heparin™ affinity chromatography (26) combined with ultracentrifugation and were then tested in the mouse model. VLPs displaying NA as described here could, in the future, be added to seasonal influenza vaccines to increase immune responses to NA.

## 2 Materials and methods

### 2.1 Cells and viruses

*Tnms*42 cells (*Trichoplusia ni* derived) free of any alphanoda virus contamination (27) and *Sf*9 (*Spodoptera frugiperda*) cells were maintained in HyClone SFM4Insect (Cytiva) supplemented with 0.1% Kolliphor P188 (Sigma-Aldrich) at 27°C. For propagation of baculovirus (BV) stocks in *Sf*9 cells, the media was supplemented with 3% fetal bovine serum (FBS, Gibco).

The challenge virus A/Switzerland/9715293/13 (H3N2) is mouse-adapted (28). The virus used in NA inhibition was A/Kansas/14/2017 (H3N2). There are 13 amino acid differences between the A/Kansas/14/2017 strain (vaccine) and the A/Switzerland/9715293/13 strain (challenge virus) in terms of NA sequence. This includes a gain of an N-linked glycosylation motif of Kansas17 in position 245/247 and a loss of a glycosylation motif relative to Swiss13 in 329/313. Both viruses were grown in 9-day-old embryonated chicken eggs (Charles River Laboratories) at 37°C for 48 h.

### 2.2 Molecular cloning and recombinant baculovirus generation

For the recombinant N2 exposed on the surface of a VLP, the nucleic acid sequence of the influenza N2 from A/Kansas/14/2017 (GenBank: MG974452.1) was synthesized as gBlock Gene (GenScript), PCR-amplified, and cloned into the pACEBac2 acceptor vector (Geneva Biotech) under control of the *AcMNPV* p10 promoter. The HIV-1 gag gene sequence (GenBank: K03455.1) was cloned into the pIDC donor vector (Geneva Biotech) (23). The two vectors were fused using Cre recombination to obtain a multigene expression vector encoding the influenza NA and the HIV-1 gag protein. A vector solely harboring the HIV-1 gag expression cassette was employed as control. The recombinant soluble NA protein used in this study was designed according to the previously reported construct N2-MPP (29), which contains an N-terminal signal peptide, followed by a hexahistidine purification tag, the MPP (measles virus tetramerization domain), a thrombin cleavage site, and the globular head of the N2 protein from A/Kansas/14/2017 (GenBank: MG974452.1, aa 77 to 471). The N2-MPP was synthesized as gBlock Gene (GenScript), PCR-amplified, and cloned into the pACEBac2 vector (Geneva Biotech) under the control of the *AcMNPV* p10 promoter. Vectors encoding the N2-MPP, only HIV-1 gag and N2-HIV-1 gag were transformed into chemical competent *E.coli* DH10EMBacY (Geneva Biotech) for Tn7-based transposition of the expression cassettes into the bacmid. To generate recombinant BV, the isolated bacmids were transfected into *Sf9* cells using the FuGene HD transfection reagent (Promega) according to the manufacturer’s instructions. The BV was propagated in *Sf9* cells to a passage 3-stock to generate higher titers.

### 2.3 Recombinant proteins and VLP expression in baculovirus-insect cell system

To express the proteins either as soluble or VLP, *Tnms42* cells were cultivated at a cell density of 2 × 10^6^ cells/mL. Cell cultures were infected with the respective BV working stock at a multiplicity of infection of three. The recombinant N2-MPP, gag-only VLP, and N2-VLP were harvested from *Tnms42* cell expression supernatants 72–96 h post-infection. The supernatant was clarified by centrifugation at 500g for 5 min, followed by another at 1000g for 10 min.

### 2.4 Purification of N2-MPP and N2-VLP

The soluble N2-MPP protein was purified by gravity-assisted flow purification protocol in disposable columns (30). Briefly, the cleared supernatant was incubated with Ni^2+^ nitrilotriacetic acid (NTA) resin (Qiagen) for 2–4 h at room temperature (RT) while shaking. The resin-supernatant mixture was then passed over 10 mL polypropylene columns (Qiagen) and washed 4x with 15 mL of washing buffer (50 mM Na_2_HCO_3_, 300 mM NaCl, 300 mM NaCl, 20 mM imidazole, pH 8). Finally, the protein was eluted with 8 mL of elution buffer (50 mM Na_2_HCO_3_, 300 mM NaCl, 300 mM NaCl, 300 mM imidazole, pH 8). The protein was concentrated using an Amicon Ultracel-15 centrifugal filter with a cut-off of 30 kDa (Millipore) and the buffer was exchanged to phosphate buffered saline (PBS) pH 7.4. For the VLPs, a platform purification method (26) was combined with ultracentrifugation to concentrate the material. The clarified supernatant was filtered with a 0.8 µm sterile polyvinylidene difluoride (PVDF) syringe filter (Thermo Fisher Scientific). For removal of host cell DNA, a medium-salt active nuclease (M-SAN; ArticZymes) treatment was performed (31) according to the manufacturer’s instructions. For pre-purification, a column packed with Capto™ Core 700 resin (Cytiva) was employed. The supernatant clarified, filtered, and treated with endonuclease was loaded onto the column previously charged with buffer A (50 mM 4-(2-hydroxyethyl)-1-piperazineethanesulfonic acid (HEPES), 2 M NaCl, pH 7.2) and equilibrated with 5% of the same buffer (equivalent to 100 mM NaCl). The flow-through was collected and loaded into a column packed with Capto™ Heparin, pre-charged with buffer A, and equilibrated with 5% of the buffer A. The Capto™ Heparin column was washed with 3 column volumes (CV) of 100 mM NaCl, and elution was performed by applying a linear salt gradient from 100 mM to 2000 mM NaCl over 20 CV in order to separate VLPs from other particles. The chromatography was performed with an ÄKTA Pure 150 M2 instrument equipped with a sample pump S9 and a fraction collector F9-C (GE Healthcare). VLPs were then concentrated via ultracentrifugation at 70,000 x g for 2 h using an SW28 rotor (Beckman). The protein concentration of the soluble protein was quantified using the Quickstart Bradford Dye Reagent (Bio-Rad) with a bovine serum albumin (BSA) standard curve. The protein concentration of the N2 on the VLPs was determined by comparing a dilution of VLPs with a known concentration of a recombinant N2 protein. Particle concentration was determined by nanoparticle tracking analysis (NTA), with a NanoSight NS300 (Malvern Instruments Ltd.) alongside a 488 nm laser module.

### 2.5 Double-stranded DNA content and infective particle titer

The contamination on supernatants and purified VLPs was determined as follows. Double-stranded DNA (dsDNA) was determined by Quant-iT PicoGreen™ dsDNA kit (Life Technologies) according to the manufacturer’s instructions. The plate was read in a GeniusPro Plate Reader (Tecan). Quantification of infective BV titer was performed by tissue culture infective dose 50 (TCID_50_) on *Sf9* cells. *Sf9* cells in early/mid-log phase were dispensed into a 96-well plate at a concentration of 0.4 x10^5^ cells per well. Samples were analyzed in triplicates and three-fold serial dilutions were performed. A volume of 25 µL of each virus dilution was added to the plates with the attached *Sf9* cells and then incubated for 5 days at 27°C. Plates were examined with a Leica DM IL LED Inverted Laboratory Fluorescence Microscope (Leica Microsystems). A modified equation from Reed and Muench was used for calculating the TCID_50_ and a Poisson distribution-derived factor was employed for conversion to pfu (32).

### 2.6 Sodium dodecyl sulfate-polyacrylamide gel electrophoresis (SDS-PAGE) and western blot

Protein identity was assessed by western blot. Samples were separated on an SDS-PAGE under reducing conditions. For the bio-sulfosuccinimidyl substrate (BS3, Thermo-Fisher) crosslinker, the proteins were treated with the crosslinker according to the manufacturer’s instructions. For the SDS-PAGE 5 µL of the respective purified samples were mixed 1:1 with 2 x Laemmli sample buffer (BioRad) supplemented with 5% 2-mercaptoethanol (Sigma). The samples were heated at 95°C for 15 min prior to loading on the SDS-PAGE gel (4–20% Mini-PROTEAN TGX™ Precast Protein Gels, BioRad). Protein bands in the gel for the SDS-PAGE were stained with Flamingo fluorescent Protein Gel Stain (BioRad). For Western Blot, after SDS-PAGE, proteins were electroblotted onto PVDF membranes (Invitrogen) which were subsequently blocked overnight (ON) at 4°C with 3% milk (AmericanBio) in phosphate-buffered saline with 0.1% Tween (PBS-T). Membranes were incubated for 1 h at RT with the corresponding primary antibody: rabbit anti-influenza A virus H3N2 NA primary antibody (Invitrogen); rabbit anti-baculovirus vp39 (ProteoGenix); mouse anti-HIV-1 p24 (abcam); rabbit anti-Histone H3 (abcam); diluted 1:1000. Goat anti-rabbit IgG secondary antibody, alkaline phosphatase (AP) conjugated (Thermo-Fisher) or goat anti-mouse IgG secondary antibody, alkaline phosphatase (AP) conjugated (Thermo-Fisher) was added at a 1:30,000 dilution. Finally, bands were visualized with a premixed nitro-blue tetrazolium chloride/5-bromo-4-chloro-3’-indolyphosphate p-toluidine salt (NBT/BCIP) solutions (BioRad).

### 2.7 NA-activity assay

To determine the NA activity of the purified proteins, an NA-star assay (Thermo Fisher) was performed following the manufacturer’s instructions. The N2-MPP and N2-VLP proteins were diluted to a starting concentration of 1 µg in 50 µL of the provided assay buffer. Two-fold serial dilutions were performed and then incubated at 37°C for 20 min. Then, the substrate was added, and the plate was incubated for 30 min at RT. To measure the chemiluminescence the accelerator solution was added, and the plate was immediately read using a Synergy H1 Hybrid multimode microplate Reader (BioTek). For quantitation of neuraminidase activity, the signal was divided by noise (value from negative controls) and signal/noise value versus enzyme amount were plotted.

### 2.8 Immunoelectron microscopy

For particle visualization, samples were prepared as described in Hausjell et al. (2023) (33). Briefly, formvar-coated copper grids were floated on VLP suspension diluted 1:50 in particle-free water. Following fixation with 2% glutaraldehyde, samples were floated on blocking buffer (5% BSA in 0.1 M phosphate buffer; pH 7.4) for 10 min. Grids were then incubated on a rabbit anti-Influenza A H3N2 NA primary antibody (Invitrogen) diluted 1:50. Antigen-antibody reaction was visualized with a donkey anti-rabbit IgG antibody conjugated with 10 nm gold (Abcam) diluted 1:50. For double labelling, grids were incubated on the same rabbit anti-influenza A H3N2 NA primary antibody and a mouse monoclonal antibody against HIV-1 p24 (abcam) diluted 1:50. As secondary antibodies to detect mouse anti-p24, donkey anti-mouse conjugated with 6 nm gold particles (Jackson immuno 715–194-150) was used. Subsequently, negative staining was performed with uranyl acetate replacement stain (UAR_EMS Stain, EMS-Electron Microscopy Sciences). For visualization, a Tecnai G2 transmission electron microscope operating at 200 kV was used.

### 2.9 Animal work

All animal experiments were carried out under protocols approved by the Icahn School of Medicine at Mount Sinai Institutional Animal Care and Use Committee. For all animal experiments, female 6–8-week-old DBA/2J mice (Jackson laboratories, n=5 per group) were used. Mice were intramuscularly vaccinated in a prime/boost regimen separated into the following groups: 1.0 µg of N2-MPP, 1.0 µg or 0.3 µg of N2-VLP, 3.0 µg of an irrelevant protein (BSA) or 2 x 10^9^ only gag-VLPs (particle amount equivalent to 1 µg of the N2-VLP vaccination) three weeks apart. Mice were then challenged with 25x mLD_50_ of A/Switzerland/9715293/2013 (H3N2, mouse-adapted). Weight loss and survival were monitored over 14 days. Blood was obtained on day 21 and day 49 after prime immunization. Mice were humanely euthanized if they lost 25% or more of their initial body weight.

### 2.10 Enzyme-linked immunosorbent assay (ELISA)

Highly binding polystyrene 96-well plates (Microwell™ MaxiSorp™ flat bottom plates, Sigma-Aldrich) were coated with 50 µL/well of the N2 recombinant protein at a concentration of 5 µg/mL in PBS (pH 7.4; Gibco) ON at 4°C. The next day, the coating solution was removed, and the plates were blocked with 200 µL/well of blocking buffer consisting of 3% goat serum (Sigma-Aldrich), and 0.5% milk (AmericanBio) in PBS-T for 1 h at RT. The blocking solution was removed, and the serum collected on d21 and d49 was added. The serum was diluted 1:50 in PBS, serially diluted 1:3, and then incubated for 2 h at RT. After serum incubation, the plates were washed 3x with PBS-T and incubated with the secondary antibody, anti-mouse IgG horseradish-peroxidase (HRP) (31430, Invitrogen). The secondary antibodies were diluted 1:3,000 in blocking buffer and added to the plate (50µL/well) for 1 h at RT. The plates were washed 3x with PBS-T, and 100 µL/well of SigmaFast o-phenylenediamine dihydrochloride (OPD) developing solution (Sigma-Aldrich) was added for 10 min. The reaction was stopped by adding 50 µL/well of 3 M hydrochloric acid (HCl). The plate was read using a Synergy H1 Hybrid multimode microplate Reader (BioTek) at an optical density of 490 nm. The data were analyzed using GraphPad Prism 10.2.0.

### 2.11 Enzyme-linked lectin assay (ELLA) to determine neuraminidase Inhibition (NI)

To test if vaccination with N2-MPP and N2-VLP induces serum antibodies with NI activity, an ELLA assay was performed as described before (34). Briefly, highly binding polystyrene 96-well plates (Microwell™ MaxiSorp™ flat bottom plates, Sigma-Aldrich) were coated with 100 µL/well of 25 µg/mL fetuin (Sigma-Aldrich) at 4°C ON. Serum samples were heat-inactivated for 1 h at 56°C and then diluted 1:50 in sample diluent which consisted of 5% BSA in Dulbecco’s phosphate-buffered saline (DPBS) complemented with calcium and magnesium (Gibco) and 2% Tween. The samples were then serially diluted 1:2 on a separate 96-well plate and 50 µL/well was added to the fetuin-coated plates. The influenza virus A/Kansas/14/2017 was diluted in sample diluent and added to the serum dilutions at 2x the 50% effective concentration (EC_50_). The serum-virus mixture was incubated for 16–18 h at 37°C. The plates were washed 3x with PBS-T and then incubated with 100 µL/well of 50 µg/mL peanut agglutinin (PNA) conjugated to HRP (Sigma-Aldrich) for 2 h at RT. The plates were washed 4x with PBS-T, and 100 µL/well of SigmaFast OPD developing solution was added. After 10 min of incubation in the dark at RT, the reaction was stopped by adding 50 µL/well of 3 M HCl. The OD was measured in a Synergy H1 Hybrid multimode microplate Reader (BioTek) at an optical density of 490 nm. The individual inhibitory dose 50 (ID_50_) was calculated using GraphPad Prism 10.2.0.

### 2.12 Statistical analysis

Titers were compared using a one-way analysis of variance corrected for multiple comparisons. Survival was compared using a Mantel-Cox log-rank test and corrected for multiple comparisons. IgG levels were compared by determination of the area under the curve. All statistical analyses were performed in GraphPad Prism 10.2.0.

## 3 Results

### 3.1 N2-VLPs set up correctly folded structures and active neuraminidases

**Figure 1:**
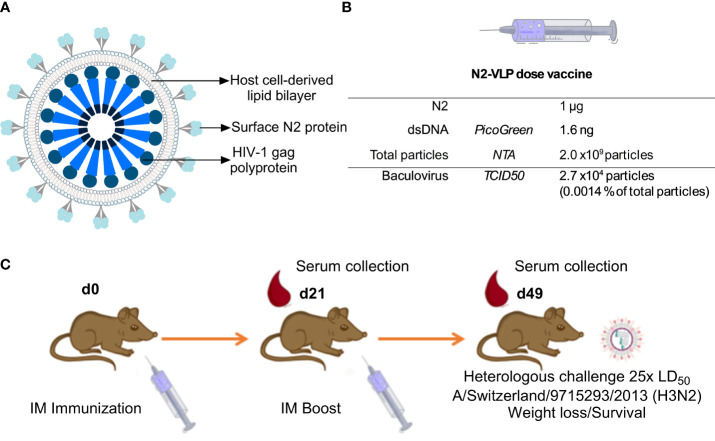
Immunization. **(A)** Schematic representation of the N2-VLP **(B)** Composition of a N2-VLP vaccine dose and the respective analytical assays employed for given quantification **(C)** Vaccination scheme for assessing the protective potential of the N2 as soluble protein or displayed at the surface of a bionanoparticle *in vivo* without adjuvant. Female 6- to 8- week-old DBA/2J mice were vaccinated in a prime/boost regimen and then challenged with 25x the LD_50_ of A/Switzerland/9715293/2013 (H3N2, mouse-adapted) and monitored for 14 days. IM: Intramuscular. Mice were bled on day 21 (d21) and day 49 (d49) for serological analysis.

To express the N2 neuraminidase on the surface of VLPs, the full sequence coding for the N2 cytoplasmic tail, the transmembrane domain, the stalk, and the NA head of A/Kansas/14/2017 were cloned into a pACBac2 vector and then fused with a pIDC-gag vector that contained the HIV-1 gag matrix protein using Cre recombination. Gag-based VLPs displaying the N2 were expressed using the BEVS and the *T.ni* insect cell line, *Tnms42 (* 35). NA-VLPs were then purified via a platform downstream procedure based on a Capto-Core 700™ flow-through chromatography followed by a Capto-Heparin™ affinity chromatography (26). The flow-through material from the Capto-Core 700 ™ was loaded onto the heparin column to separate the gag-VLPs from baculovirus particles and other extracellular vesicles. An immediate breakthrough of particles during loading followed by two overlapping peaks during elution was observed at the chromatogram of this purification step (**Supplementary Figure 1A**). To corroborate that particle populations were separated as Zollner et. al., reported, protein composition was determined by SDS-PAGE and Western blots (**Supplementary Figures 1B–E**). HIV-1 p24 Western blot (**Supplementary Figure 1B**), can be found with higher intensities in peak 1 (P1 or E2); the capsid protein of the baculovirus vp39 (**Supplementary Figure 1C**) is enriched in the second peak (P2 or E4 and E5). An H3 histone antibody was employed as an indicator for the presence of chromatin (**Supplementary Figure 1D**), the Western blot showed that it was removed by the Capto-Core 700 ™ step. Finally, the purification of the sample was corroborated by SDS-PAGE analysis, the P1, which corresponds to the VLP enriched fraction showed fewer bands than the loading material, and the supernatant of the *Tnm42* cells that was employed to search for host cell protein (**Supplementary Figure 1E**). The VLP enriched fraction was then concentrated by ultracentrifugation to a NA concentration of 0.05 mg NA/mL. This concentration of NA was equal to ~1 x10^11^ particles/mL. Contaminants such as baculovirus (BV) and double-stranded DNA (dsDNA) were determined by tissue culture infective dose 50 (TCID_50_) and PicoGreen™ assay respectively. Raw supernatants contained 1.6 x10^3^ ng/mL dsDNA while after purification the samples only contained 80 ng/mL of dsDNA. This corresponded to 1.6 ng/dose (**Figure 1B**) which meets the requirements of the regulatory agencies with <10 ng of residual dsDNA per dose (36). Moreover, the amount of BV particles decreased from 2.7 x10^7^ plaque-forming units (pfu)/mL in raw supernatants to 1.4 x10^6^ pfu/mL of BV in the final product fraction, which corresponded to 2.7 x10^4^ pfu BV per dose (**Figure 1B**). A recombinant version of the head domain of the same N2 fused to an N-terminal signal peptide, hexahistidine tag and measles virus phosphoprotein tetramerization domain (N2-MPP) (29, 37) was expressed in the same system and purified via Ni^2+^ affinity chromatography. This protein served as a comparator for the following experiments.

**Figure 2:**
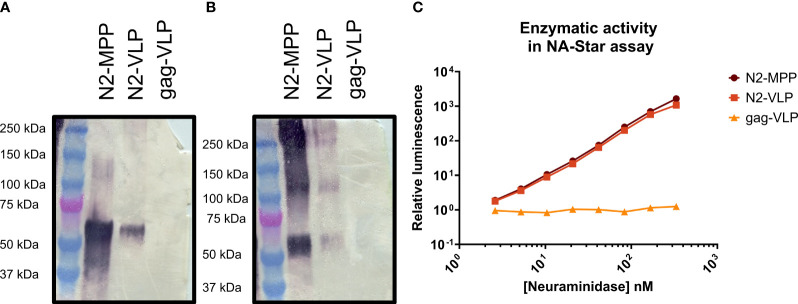
Analysis of N2-MPP and N2-VLP. **(A)** Western blot under reducing conditions using a polyclonal antibody against the N2. **(B)** Western blot using a BS3 cross-linker and a polyclonal antibody against the N2. **(C)** NA activity was determined via NA-Star assay. 1.0 nM NA is equivalent to 0.003 µg of NA/50 µL assay volume, based on a subunit molecular weight to 60 kDa. A gag-VLP (with no protein expressed on the surface) was employed as a negative control. This assay was run once in duplicates, geometric mean, and geometric standard deviation are shown.

To verify the protein identity and structural integrity of the recombinant proteins, the N2 was visualized by Western blot analysis using a polyclonal NA-antibody (**Figures 2A, B**). Under reducing conditions, the N2-MPP and N2-VLP showed a band at around 60 kDa, corresponding to the size of the N2 monomer (**Figure 2A**). Since only tetrameric NA and not the monomer is able to induce a protective immune response in mice (19) the proper formation of tetramers was tested by employing a BS3 cross-linker, which connects primary amines. After the cross-link reaction, the Western blot analysis showed the formation of tetramers at around 240 kDa and dimers at 120 kDa (**Figure 2B**). Correct folding of the NA was also determined by measuring the NA activity, considering that only tetrameric NA and not the monomeric is showing robust enzymatic activity (19). Both the soluble N2 and N2-VLPs exhibited high enzymatic activity (**Figure 2C**). The gag-VLPs without N2 expressed at the surface were used as negative control and did not show any NA activity.

**Figure 3:**
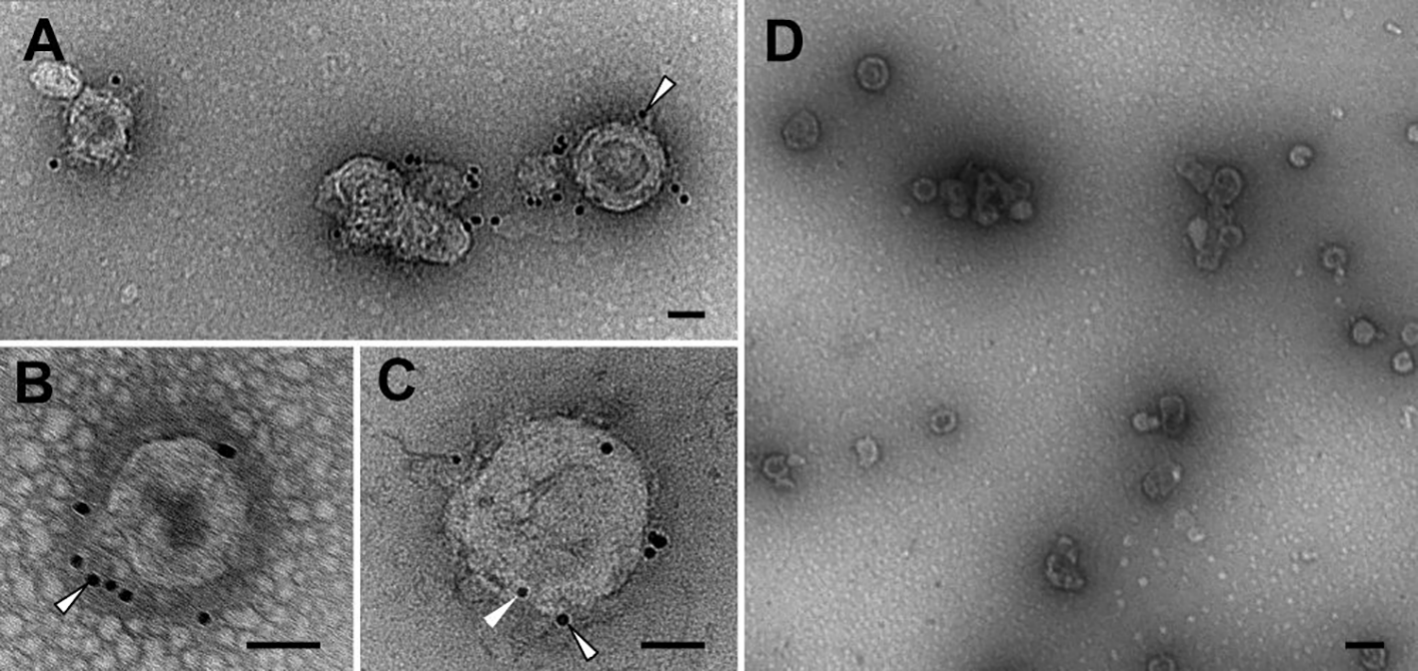
Transmission electron microscopy. Immunolocalisation of NA. Abundant VLPs and high purity level can be observed in N2-VLPs **(A–C)** and gag-VLPs **(D)** samples. See the labelling for N2 on the N2-VLPs (**A–C**, outlined arrowheads), as well as the presence of p24 on the N2-VLPs (**C**, arrowhead) and the absence of significant labelling on the negative control **(D)**. Bars 50 nm **(A–C)**, 200 nm **(D)**.

Transmission electron micrographs (**Figure 3**) and size distribution determined by NTA (**Supplementary Figure 2C**) of the purified samples showed the presence of round-shaped particles with an average size of 100–200 nm, consistent with previous reports on gag-based VLPs (38, 39). The morphology of gag-based nanoparticles has been reported as similar to HIV-1 immature particles, in which the distribution of the exposed proteins is grouped in more than one single focus unlike mature particles (40, 41). The exposition of the N2 antigen on the surface of the VLPs was confirmed by immunolocalization of the NA by transmission electron microscopy (TEM). The sample containing the N2-VLPs showed bionanoparticles surrounded by black dots representing the gold-labeled N2 (**Figures 3A–C**), no unspecific binding was observed for the gag-only particles (**Figure 3D**, **Supplementary Figures 2A, B**).

### 3.2 Vaccination with N2-VLP provides protection against influenza virus challenge

We aimed to test the N2 incorporated into VLPs (**Figure 1A**) without adjuvant in terms of potency as vaccine in a mouse study. The VLPs were compared to soluble NA which, when adjuvanted, induces robust protection against challenge (29). Therefore, six-to-eight-week-old DBA/2J mice (n=5 per group) were vaccinated in a prime/boost regimen. Mice were intramuscularly primed with 0.3 or 1.0 µg N2 NA protein without adjuvant, either using the soluble immunogen or NA-VLPs, and were boosted three weeks later using the same dosage. The composition of the novel influenza vaccine candidate is indicated in **Figure 1B**. A group vaccinated with equivalent particle composition but no N2 exposed at the surface was employed as control (gag-VLP dose: 2 x10^9^ particles, 2.7 x10^4^ particles and 4.1 ng of dsDNA). Four weeks post booster immunization, mice were intranasally challenged with 25x the 50% mouse lethal dose (mLD_50_) of the mouse-adapted heterologous H3N2 strain A/Switzerland/9715293/2013 and monitored for 14 days (**Table 1**, **Figure 1C**).

**Table 1:** Vaccination regimen for N2-VLPs and N2-MPP as a standalone vaccine.

Group	Prime	Boost
1	0.3 µg N2 on the surface of VLPs	0.3 µg N2 on the surface of VLPs
2	1.0 µg N2 on the surface of VLPs	1.0 µg N2 on the surface of VLPs
3	1.0 µg N2-MPP	1.0 µg N2-MPP
4	gag-VLP equivalent number of particles of 1.0 µg N2-VLPs	gag-VLP equivalent number of particles of 1.0 µg N2-VLPs
5	3.0 µg irrelevant BSA	3.0 µg irrelevant BSA

**Figure 4:**
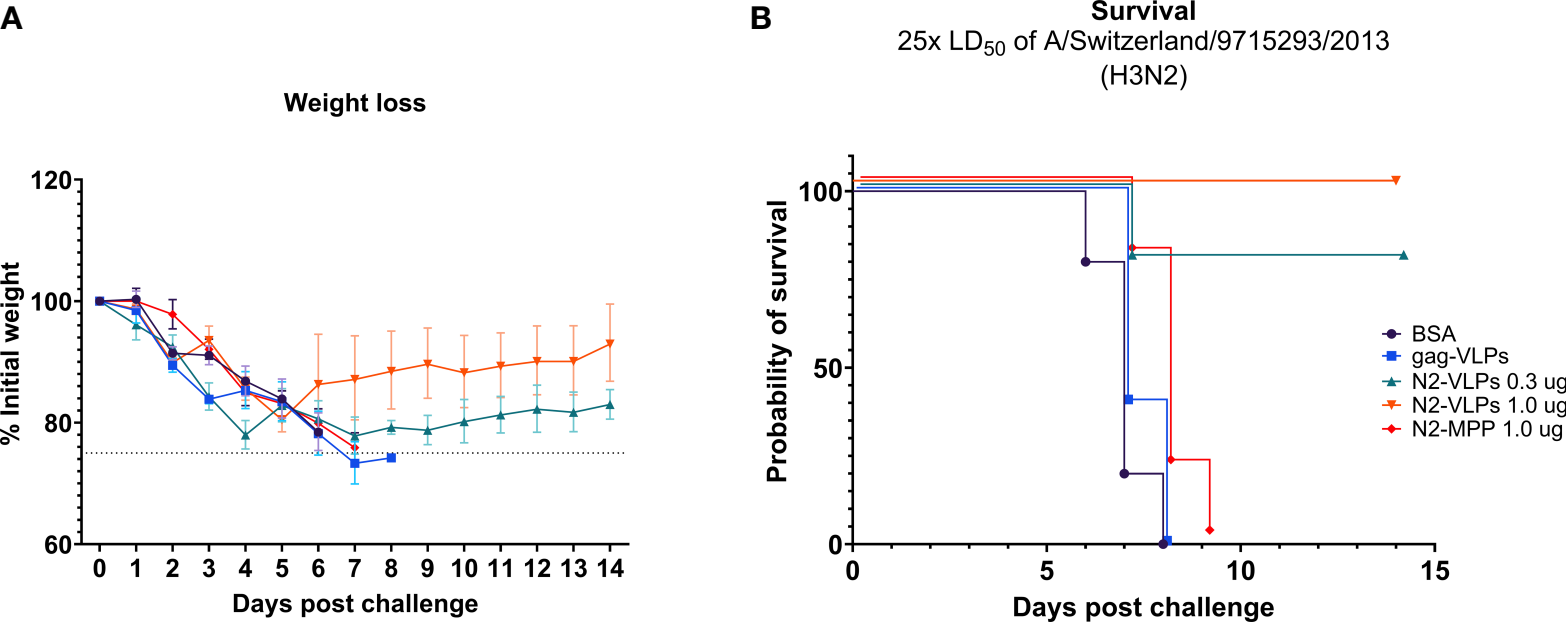
Protective potential of N2-VLP as vaccines *in vivo*. Weight loss curve **(A)** and survival curve **(B)** after challenge. n=5. **(A)** The dotted line indicates 75% of the initial weight, the endpoint for weight loss. Geometric mean and geometric standard deviation are shown.

Mice vaccinated with 1.0 µg of the NA-VLPs were fully protected from mortality but experienced a ~15% weight loss (**Figures 4A, B**). In contrast, the group that received 1.0 µg of the soluble unadjuvanted NA (N2-MPP) succumbed to the lethal infection between days 7 to 9 (**Figures 4A, B**). The group that received 0.3 µg of N2 on VLPs showed 20% weight loss, with one mouse succumbing to infection on day 8 post-challenge (**Figures 4A, B**). N2-VLP vaccinated groups showed moderate weight loss, with some intermediate recover from day 3 to day 5 post-challenge, and they finally fully recovered. We believe that these minor differences are an artifact due to small group sizes. Differences in survival were analyzed using a Mantel-Cox log-rank test corrected for multiple comparisons: BSA vs N2-VLP 1.0 µg (p=0.002); gag-VLP vs N2-VLP 1.0 µg (p=0.0023); N2-VLP 1.0 µg vs N2-MPP 1.0 µg (p=0.0020); were statistically significant. Other differences were not statistically significant (ns; Bonferroni-corrected α value for 10 comparisons p>0.005).

In summary, in a prime/boost regimen, 1.0 µg of the N2 displayed on the surface of a VLP provided 100% protection from mortality post-challenge. A statistical difference in the probability of survival was observed by comparing this group to the group that was vaccinated with 1.0 µg the N2-MPP protein (p<0.005).

### 3.3 Vaccination with N2 VLPs results in robust induction of anti-NA antibodies

**Figure 5:**
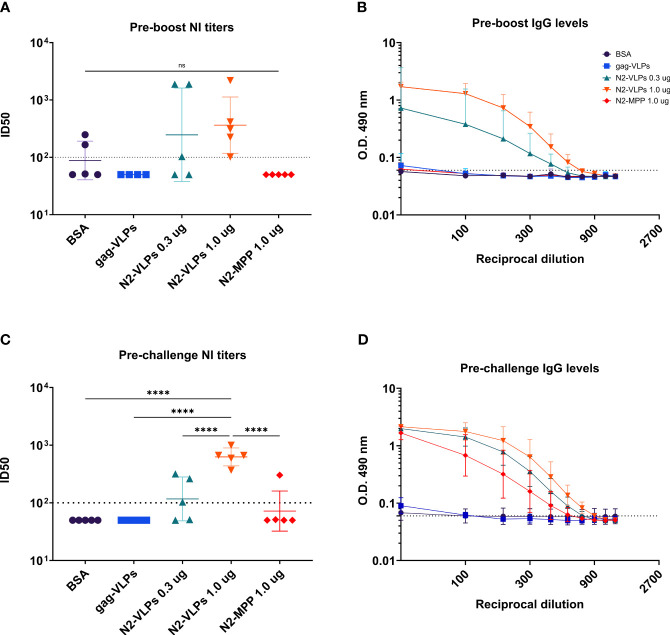
Serological assessment of serum samples obtained after N2 vaccination. Serum samples were collected before the boost and before the challenge. **(A)** NI assay using an A/Kansas/14/2017 H3N2 virus. Serum obtained before the boost. **(B)** ELISA against recombinant N2 A/Kansas/14/2017 NA before the boost. **(C)** NI assay using a homologous A/Kansas/14/2017 H3N2 virus. Serum obtained before the challenge. **(D)** ELISA against recombinant N2 A/Kansas/14/2017 NA before the challenge. **(A, C)** Negative samples were set to half of the limit of detection for graphing and analysis purposes. The dotted line represents the limit of detection. **(B, D)** Geometric mean and geometric standard deviation are shown. The AUC value was determined to compare the reactivity of the serum to recombinant N2 protein. ****, p-value <0.005.

To analyze the characteristics of serum antibodies obtained after vaccination with the different constructs, their capacity to bind to recombinant N2 protein was tested. Serum was obtained three weeks after priming as well as prior to challenge (**Figure 1C**). Before the boost vaccination (1^st^ bleed), strong antibody response was detected only in the groups vaccinated with the N2-VLPs (**Figure 5B**). The strongest response was detected in the group vaccinated with 1 µg, the calculated area under the curve (AUC) was 3.852 as compared to 2.073 of the group vaccinated with only 0.3 µg. The same trend was seen in the neuraminidase inhibition (NI) assay when an H3N2 virus from A/Kansas/14/2017 was used (**Figure 5A**), the inhibition was detectable only in the groups vaccinated with the N2-VLP. However, no statistical significance between groups was determined. After the second vaccination and before challenging (2^nd^ bleed), the antibody reactivity of the group that received 1.0 µg of the N2-MPP was markedly higher with an increase of the AUC from 0.44 to 2.76 (**Figure 5D**). Still, the group that received the N2 integrated into VLPs showed higher AUC than the group that received the same amount as N2-MPP (AUC: BSA=0.55, gag-VLP=0.50, N2-VLP 0.3 µg=4.23, N2-VLP 1.0 µg=5.70, N2-MPP 1.0 µg=2.77). Furthermore, the NI assay exhibited only a moderate gain for the group that was vaccinated with the N2 in soluble form (**Figure 5C**). The group vaccinated two times with 1.0 µg of N2-VLP produced higher NI titers and in a one-way ANOVA corrected for multiple comparison, it was the only group statistically different from all other groups (p<0.005).

## 4 Discussion

Conventional influenza virus vaccines lower the risk of disease in the general population by 40% to 60% (42). These vaccines are based on influenza virus HA as a major antigenic component and rely on the induction of HA-specific neutralizing antibodies (1). In contrast to natural infection, seasonal vaccination lacks the ability to induce a robust immune response against the influenza virus NA (21), which is the second most abundant viral glycoprotein. A potential solution to this problem is to supplement current vaccines with correctly folded, stable, tetrameric NA protein or alternatively with bionanoparticles that display tetrameric NA on an outer membrane. It was shown that vaccines incorporating standardized amounts of NA have the potential to provide broader and longer-lasting protection against influenza virus infection (16–21). In this study, we compared the efficacy of NA vaccination in a mouse model administered either as soluble protein or displayed on the surface of VLPs.

Subtypes A(H1N1) and A(H3N2) of the influenza virus are currently circulating in the Northern hemisphere (season 2023/24). Vaccination against influenza generally provides reduced protection against influenza A (H3N2) strains as compared to other influenza strains (43). Here, the N2 from A/Kansas/14/2017 (H3N2) has been chosen as vaccine antigen. Heightened anti-N2 immunity might offer additional protection against H3N2, especially in years where the HA between vaccine strain and circulating strain is mismatched. In addition, some protection from strains with pandemic potential may be expected if they possess a N2 neuraminidase subtype (such as H2N2, H5N2, H7N2, or H9N2).

Recombinant N2 VLPs were generated using the BEVS and a novel *T. ni* insect cell line (*Tnms42*) (35). This was compared to expression of a soluble tetrameric version of the same N2. The soluble protein’s volumetric yield after concentration was 0.6 mg/L culture, as expected for a soluble NA produced on an insect cell system (30). The target antigen content on the produced VLPs was 0.067 mg N2/L culture. Since the presence of NA on VLPs has received little attention in the past, we could only compare it to the 3mg HA/L output from previous production processes of HA-VLPs (24). This is consistent with reports showing that NAs express at lower levels than HAs (30). Moreover, NA-VLPs underwent a two-step chromatography purification process, which has previously been successful in producing high-purity VLPs (26). Semi-purified VLP material has been used up to this point, which may have caused an overestimation of the antigen’s quantification because extracellular vesicles (EVs) and BVs share the same cell membrane and exhibit proteins on the surfaces that are similar to those of VLPs, such as NA or HA.

A prior study reported the use of a recombinant N2 linked to the measles virus phosphoprotein tetramerization domain (N2-MPP) to provide protection in a mouse model, especially when adjuvanted. However, here we wanted to assess and compare nonadjuvanted antigens. The use of adjuvants in humans, which may be needed for a purified recombinant protein vaccine, may increase reactogenicity. Vaccine candidates that can be used without adjuvant are therefore preferred. One way to increase immunogenicity without using adjuvants is to present antigens repetitively on particles like the N2 VLPs developed here. In this study, we showed that the presence of correctly folded, tetrameric NA on VLPs accurately mimics the morphology of infectious influenza virions (100–200 nm round-shaped structures displaying dispersed NA spikes on their surface determined by TEM and NTA) and confers robust protection. In a prime/boost vaccination, mice that received 1µg of NA displayed on VLPs were completely protected from mortality, performing significantly better than the nonadjuvanted soluble N2. For both NA vaccines used in this study, it is still unclear whether raising the dose might result in enhanced protection. It is worth mentioning that multiple studies have shown more robust immunity to NA of subtype N2 than to N1 in humans (17, 34, 44), suggesting a possible wider range of protection with our vaccine. Heterologous or heterosubtypic cross-protection of this N2-VLP vaccine needs to be explored more extensively in the future. However, we would like to note that the challenge strain was heterologous to the vaccine strain and yet, we did see good protection.

VLPs have previously been shown to induce specific humoral and cellular responses (22, 45, 46). In this study, neuraminidase inhibition (NI) antibodies were elicited after a single immunization with N2-VLPs. More importantly, after the boost immunization, all the mice vaccinated with two doses of 1.0 µg N2-VLPs reached the highest concentration of NI titers confirming that the NA-VLP vaccine consistently outperformed the recombinant soluble nonadjuvanted NA vaccine. Interestingly, two mice vaccinated with 0.3 µg of N2-VLP were protected without generating detectable NI. Since the limit of detection for NI titers is quite high it might be possible that sub-detectible levels of these NI titers could confer protection in those mice. Alternatively, the vaccine might be associated with other immunological mechanisms that allow protection from challenge. For example, antibodies that recognize antigenic regions of the NA that are not critical for NAI but important for monoclonal antibody binding or neutralization activity (28). More research is required to fully understand the immunological response triggered by this vaccine. On the other hand, NA-specific antibodies were generated against the N2-MPP, and in one mouse vaccinated with two doses of 1 µg of the N2-MPP, NAI antibodies were elicited, but not at a level high enough to protect. Antibodies that bind but do not neutralize the virus and thus, have no protective function *in vivo* are nearly universally elicited after infection or vaccination. In addition, VLPs may trigger a better T-cell response which may also contribute to protection.

The presence of BV contamination in the VLP vaccine derived from insect cells is another reason linked to the robust immunogenicity of the N2 VLPs. Abe et al., reported that inoculation with a dose of 10^7^ pfu of empty BV induces innate immunity that conferred nonspecific antiviral activity to mice (47). Despite the fact that the purification technique we used was successful in separating VLPs from other particles, the highest dose of VLP contained 2.7 x10^4^ BV particles, probably conferring some level of the beneficial effect that Abe et al. (47) reported. BV contamination has been known for a long time, because of the structural resemblance to the VLPs (46). Prior research has indicated that experimental vaccines contain up to 4.5 x10^6^ pfu BVs per vaccine dose, which can account for up to 5% of the total protein content (48–50). When using the baculoviral expression vector system for mammalian cell transduction, a generally 20- to 40-fold less BV is found in the expression supernatant. Yet, also VLP yields are lower compared to BEVS-based expression in insect cells (51, 52). Our methodology achieves a 150-fold BV reduction maintaining VLP yields from BEVS-based expression in insect cells. Safety is the primary consideration for vaccines, the BV is biologically harmless for humans because the BV cannot integrate its DNA into the host genome in the absence of selective pressure (53). The general safety for the application of BVs in vaccine production and human cell therapy has been the focus of attention in diverse studies and documents of the Organization of Economic Co-operation and Development (OECD). According to them, the BVs have no negative effects on human health, and there is no evidence of carcinogenicity, genotoxicity, or reproductive toxicity (54). Up to now, more than 10 BEVS-derived vaccines have been approved for veterinary and human use and, a growing number of BEVS derivatives have entered preclinical or clinical phases (55). Vaccines for human use include Flublok® (Sanofi Pasteur), Flubok Quadrivalent® (Sanofi Pasteur), Cervarix™ (GSK), NVX-CoV2373 (Novavax), Weikexin (Westvac), Trivalent Weikexin (Westvac), VidPrevtyn Beta (Sanofi/GSK) and SpikoGen® (Vaxine/CinnaGen Co.).

Although this work successfully shows protection in a mouse model, it is noteworthy that the generation of antibodies targeted at structural protein fragments from HIV-1-based VLPs remains a concern following the introduction and cancelation of the UQ-CSL v451 COVID-19 vaccination trial (ClinicalTrials.gov ID NCT04495933). It was not examined whether this vaccine candidate could cause issues with diagnostic interference. A re-engineered HIV-1 gag VLP platform or alternative pseudotyped viral particles such as the previously used severe acute respiratory syndrome coronavirus-2 (SARS-Cov2) murine leukemia virus (MLV)-gag based (56, 57) or the recently approved in Canada Covifenz® (Medicago) SARS-Cov-2 VLP (hepatitis B virus (HBV)-based VLP) (58), may be used.

In summary, we find that in the mouse model, exposure of the N2 on VLPs surface results in strong anti-NA immunity and protection from lethal influenza virus infection. On top of that, this approach might be readily adjusted to produce VLP-based vaccines to combat emerging infectious diseases; in fact, it has been demonstrated that more than 100 viral proteins from 35 different viral families can assemble into VLPs (59). This platform is a very innovative and promising approach to developing modern vaccines.

## Data availability statement

The raw data supporting the conclusions of this article will be made available by the authors, without undue reservation.

## Ethics statement

The animal study was approved by Icahn School of Medicine at Mount Sinai Institutional Animal Care and Use Committee. The study was conducted in accordance with the local legislation and institutional requirements.

## Author contributions

LR: Formal analysis, Investigation, Methodology, Writing – original draft, Data curation, Visualization, Writing – review & editing. AZ: Investigation, Methodology, Validation, Writing – original draft, Formal analysis. IH: Formal analysis, Investigation, Methodology, Writing – original draft, Data curation. EA: Investigation, Methodology, Writing – original draft, Visualization. FK: Conceptualization, Funding acquisition, Resources, Supervision, Writing – original draft. MK: Project administration, Supervision, Writing – original draft, Conceptualization, Methodology, Visualization. AJ: Conceptualization, Supervision, Writing – original draft. RG: Conceptualization, Funding acquisition, Project administration, Supervision, Writing – original draft, Writing – review & editing.
