# Supplementary material for: Increased efficacy of influenza virus vaccine candidate through display of recombinant neuraminidase on virus like particles
Source: Front Immunol. 2024 Jun 10;15:1425842. doi: 10.3389/fimmu.2024.1425842 (PMC11194364; doi:10.3389/fimmu.2024.1425842)
Supplement: Supplementary file 1 [file DataSheet_1.docx]

Supplementary Material


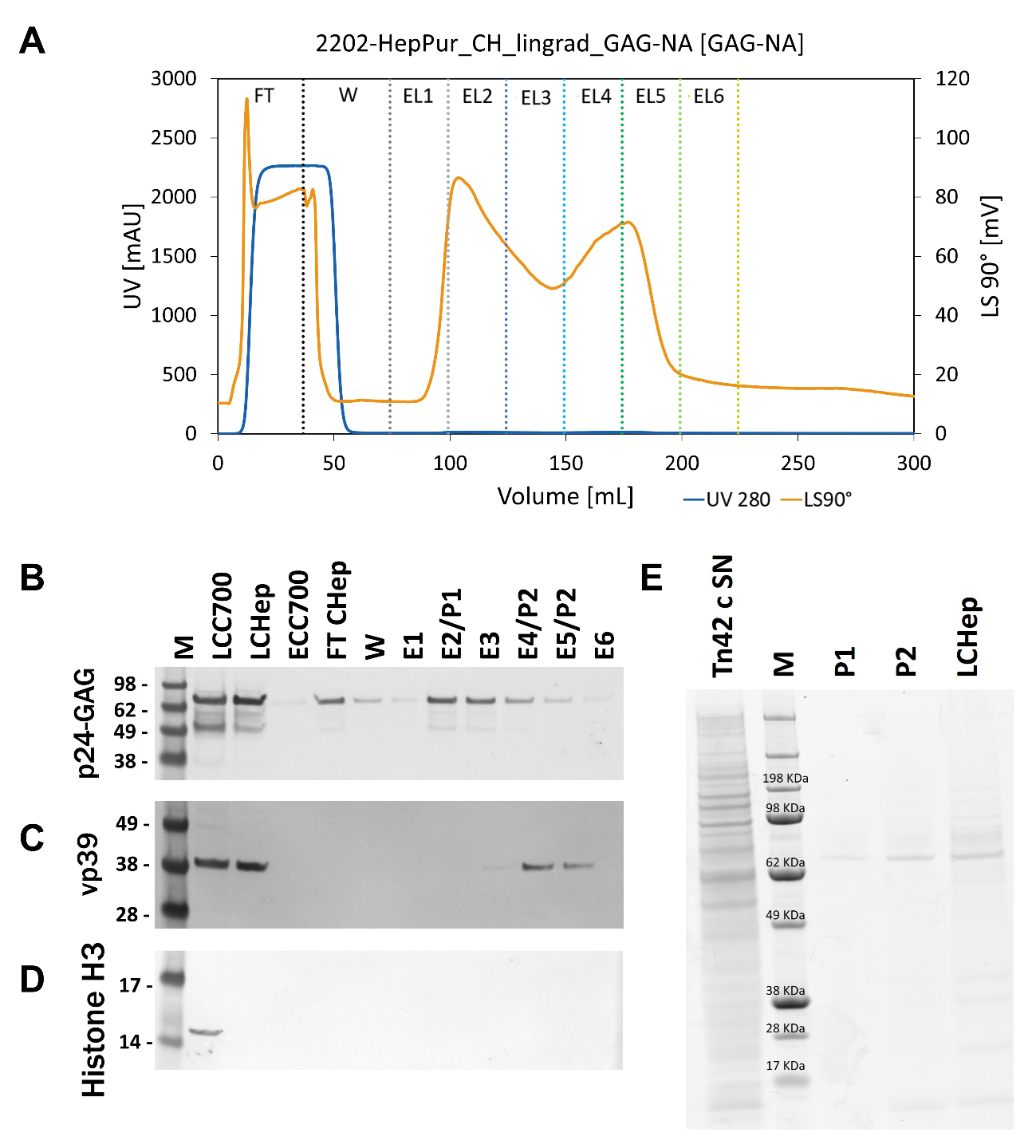


**Supplementary Figure 1: Chromatography purification.** (A) Chromatogram of the heparin affinity chromatography. The chromatogram shows the UV 280 data (blue solid line), the 90° light scattering (LS) signal (orange solid line). Relevant fractions: flow-through (FT), wash (W), elution (EL1-EL6). Purification process (B) HIV-1 p24 presence, (C) baculovirus VP39 presence, (D) histone H3 presence. (B-D) Relevant fraction: load Capto-Core 700 ™ (LCC700), load Capto-Heparin ™ (LCHep, flow-through from CC700), flow-through Capto-Heparin ™ (FT CHep), wash (W), elution (E1-E6). (E) SDS-PAGE for host cell proteins. The fractions shown are: *Tnm42* cells supernatant (*Tn42* c SN), P1 (peak 1, corresponding to EL2), P2 (peak 2, corresponding to EL4 and EL5), LCHep (Load Capto™-Heparin).


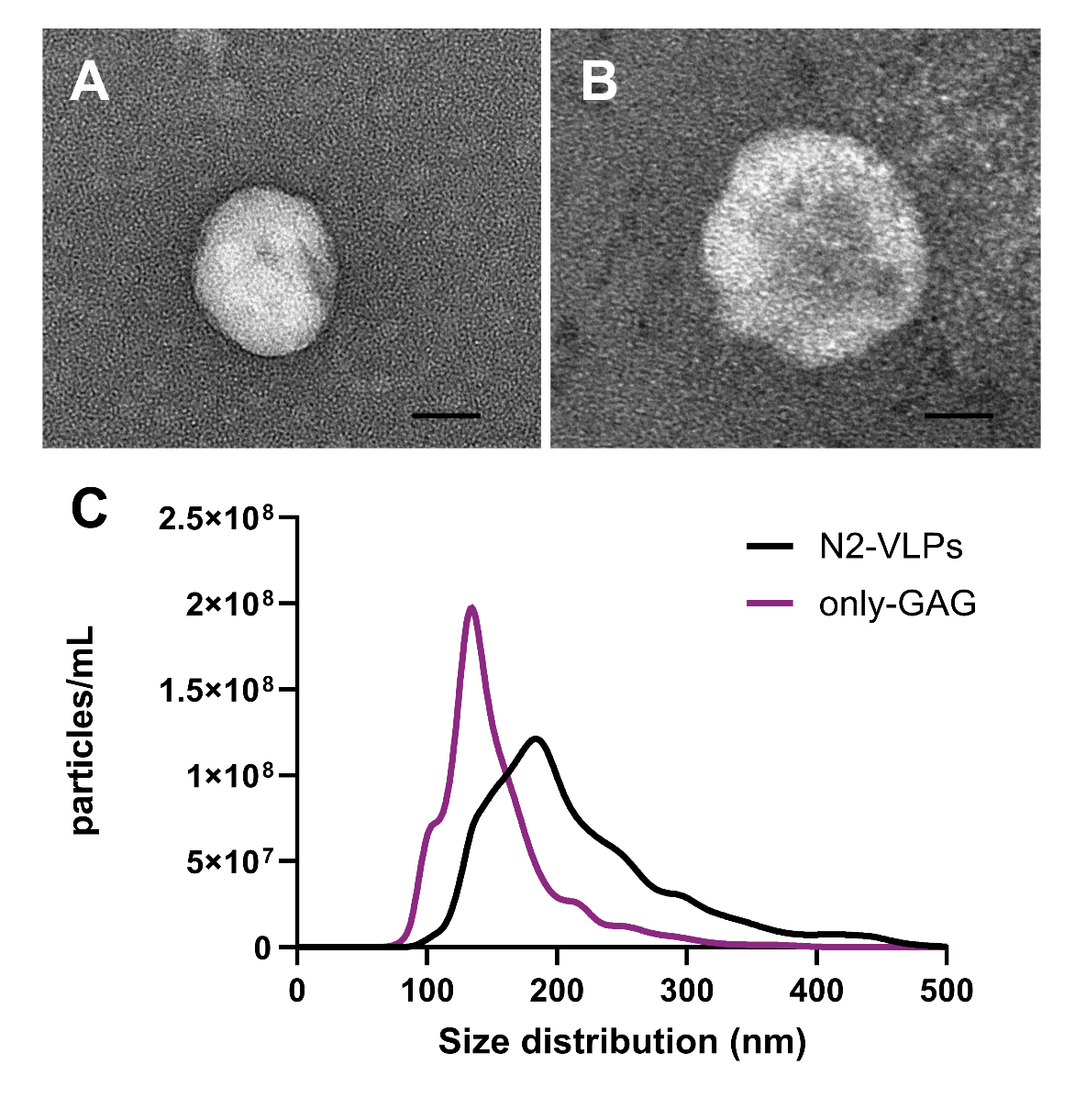


**Supplementary Figure 2: Only gag-VLPs. (A, B) Immunolocalisation of NA.** No significant labelling on gag-VLPs, representative gag-VLPs. Bars 50 nm. (C) Size distribution of the VLPs determined by nanoparticle tracking analysis (NTA).
